# Supplementary material for: Toxinome—the bacterial protein toxin database
Source: mBio. 2023 Dec 20;15(1):e01911-23. doi: 10.1128/mbio.01911-23 (PMC10790787; doi:10.1128/mbio.01911-23)
Supplement: Supplemental Figures — Figures S1-S4. [file mbio.01911-23-s0001.pdf]

Supplementary Figures

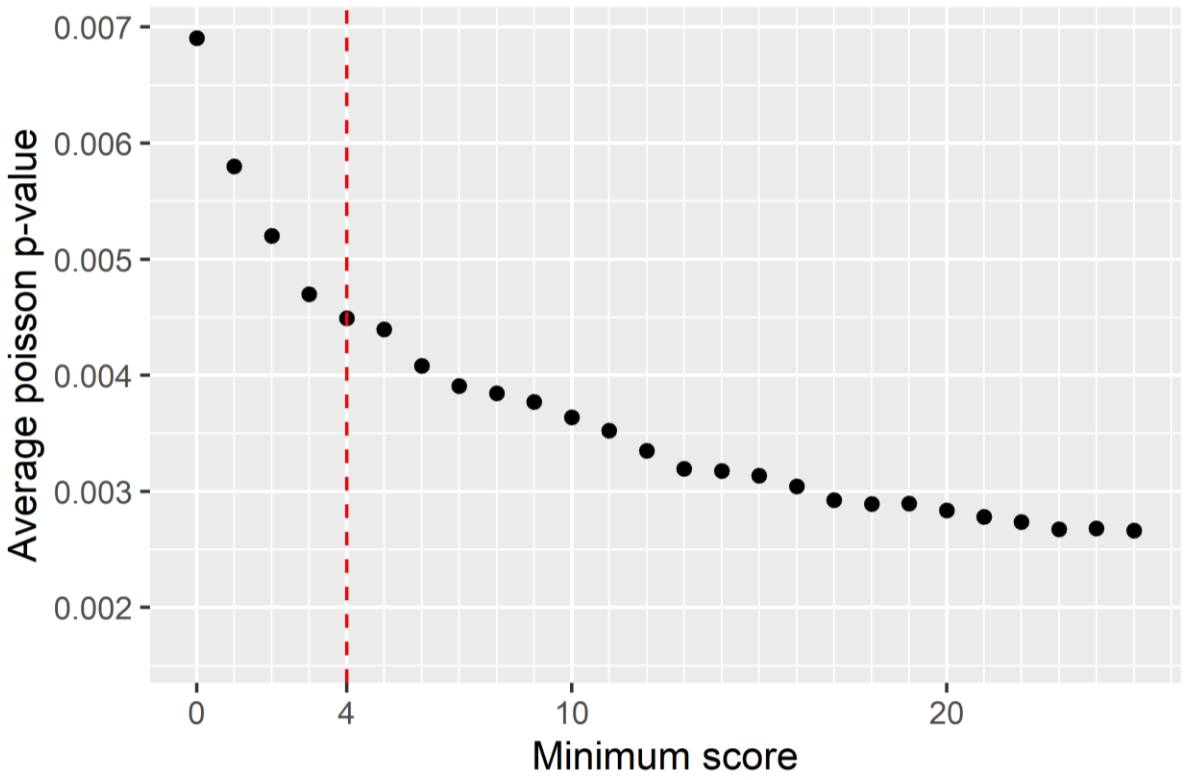

Supplementary Figure 1. Average Poisson P-value of Toxin Islands in response to incrementally increasing the minimum score threshold. The X-axis represents the minimum score cutoff of the results, and the Y-axis represents the average Poisson P-value after the cutoff. The plot illustrates how the average Poisson P-value changes as the minimum score cutoff increases. The selection of a minimum score threshold = 4 was made based on its superior improvement in the analysis.

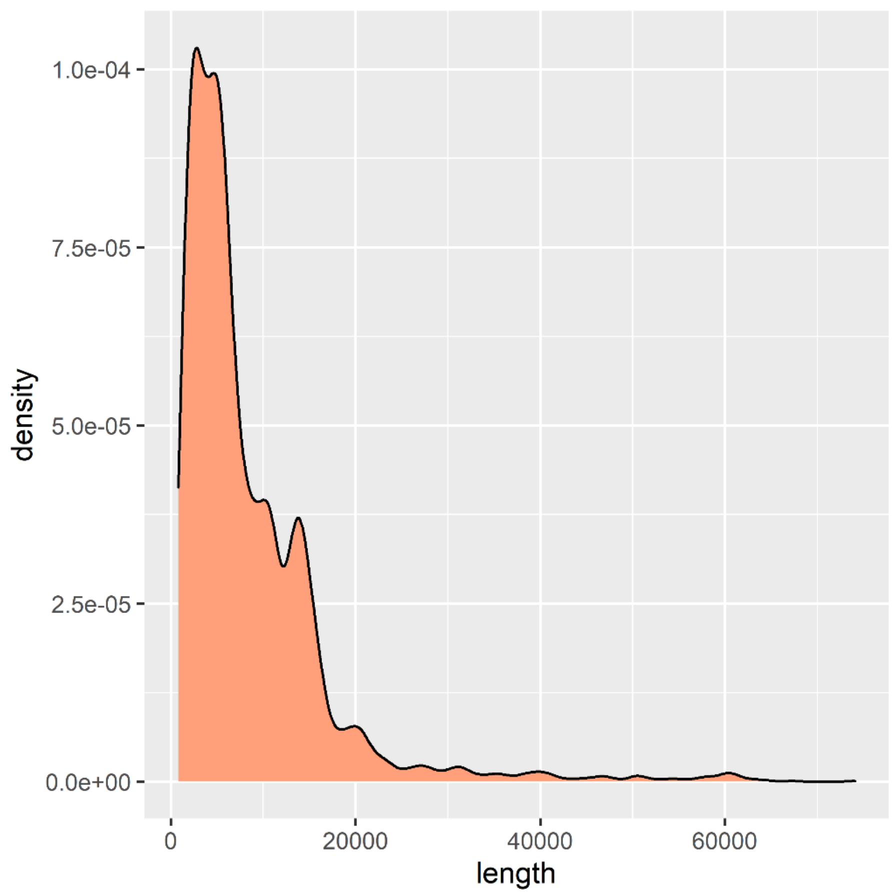

Supplementary Figure 2. Length Density of Discovered Toxin Islands. The plot represents the density distribution of unique Toxin Islands's lengths in bp, as revealed by our analysis. The X-axis represents the length pf the Toxin Islands, while the Y-axis indicates the density of the frequency of occurrence. The majority of the islands have lengths shorter than 15 Kbp, indicating a prevalence of relatively compact Toxin Islands.

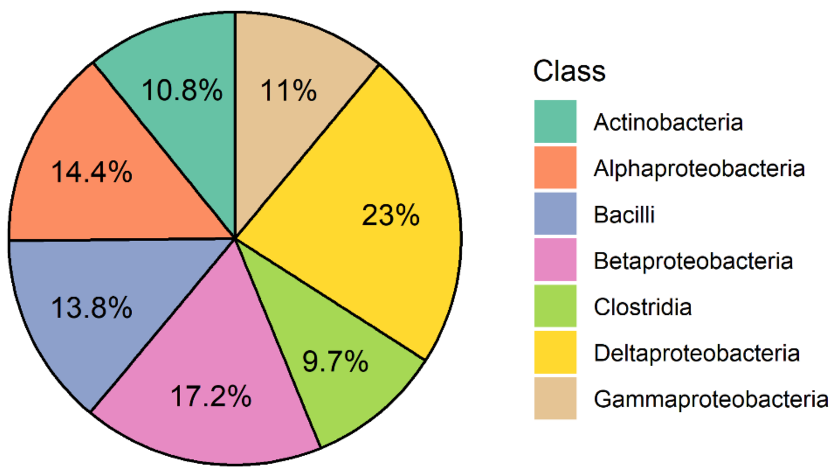

Supplementary Figure 3. Number of unique Toxin Islands identified per class normalized by the number of genomes analyzed. This plot illustrates the normalized distribution of unique Toxin Islands discovered across different classes. Each slice represents a specific class, and the size of each slice corresponds to the number of distinct Toxin Islands identified divided by the number of genomes analyzed within that class. Classes that exhibit low Toxin Island counts and genomes that lack taxonomy annotation were excluded from the analysis.

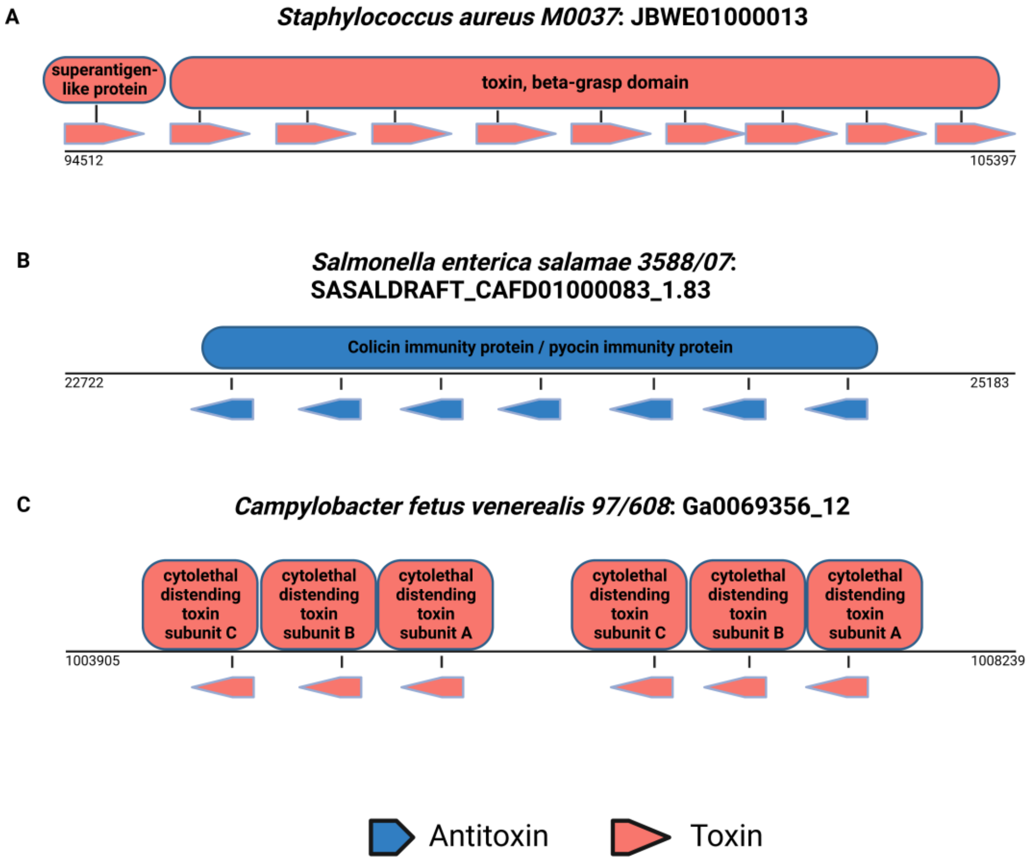

Supplementary Figure 4. Toxin Islands with homologous proteins. The figure displays three distinct examples of Toxin Islands found in different species. The title contains the contig encoding the island. The function of each protein is described above its corresponding triangle illustration in the genomes. Proteins assigned with the same function description are considered homologous proteins, likely resulting from a local duplication.
